# Prevalence and Pattern of GATA3 Immunohistochemical Expression in Female Genital Tract Adenocarcinomas

**DOI:** 10.30699/IJP.2024.2016228.3217

**Published:** 2024-01-29

**Authors:** Elham Mirzaian, Tahereh Doustmohammadi, Mahshid Panahi, Soheila Sarmadi, Fereshteh Ameli, Fatemeh Nili

**Affiliations:** 1 *Department of Pathology, Shariati Hospital, Tehran University of Medical Sciences, Tehran, Iran*; 2 *Department of Pathology, Imam Khomeini Hospital Complex, Tehran University of Medical Sciences, Tehan, Iran*; 3 *Department of Pathology, School of Medicine, Iran University of Medical Sciences, Tehran, Iran*; 4 *Department of Pathology, Yas Women Hospital, Tehran University of Medical Sciences, Tehran, Ian*

**Keywords:** Adenocarcinoma, Female genital tract, GATA3, Immunohistochemistry

## Abstract

**Background & Objective:**

GATA3 immunohistochemistry has been described as a highly sensitive marker in determining carcinomas of breast and urothelial origin. In the gynecologic system, it can be used as a marker to diagnose mesonephric or mesonephric-like carcinomas and trophoblastic tumors. The present study was performed to determine the diagnostic value of GATA3 in gynecological adenocarcinomas.

**Methods:**

A total of 187 samples from different types of endometrial, endocervical, and ovarian carcinomas were analyzed for intensity and percentage of GATA3 expression in tumor cells. The relationship between GATA3 expression and clinicopathological parameters was investigated.

**Results:**

A total of 187 patients including 101 ovarian, 77 endometrial, and 9 endocervical adenocarcinomas were investigated. Weak and focal expression of this marker was observed in 5. 1% (4/77) endometrial, 12.9% (13/101) ovarian, and 11.1% (1/9) endocervical adenocarcinomas. The mean H score in all subtypes was less than 10.6 (2-35). There was no statistically significant correlation between GATA3 expression in tumor cells with clinical stage, and tumor recurrence or metastasis.

**Conclusion:**

GATA3 is infrequently, weak, or focally expressed in most of the common gynecological adenocarcinomas.

## Introduction

Ovarian tumors are divided into epithelial, germ cells, and sex cord-stromal tumors based on their origin. Most malignant ovarian tumors are in the epithelial category and include histological types of serous, mucinous, endometrioid, clear cell, Brenner's, mesonephric, and mixed epithelial tumors (1, 2). 

Epithelial ovarian cancers, particularly high-grade serous carcinoma (HGSC), are the leading cause of death from gynecologic cancers in the United States and the fifth most common cancer diagnosed in women worldwide (3, 4). 

Endometrial cancer is the most common gynecological cancer and the fourth cause of cancer in women worldwide (5). It was histologically divided into two general categories (6). Type 1 is the most common form of the disease, which accounts for more than 70% of the cases. It is low-grade and is known as endometrioid adenocarcinoma (7, 8). Type 2 tumors are usually high-grade, serous, and clear cell histological types. These tumors have a poor prognosis with a high risk of recurrence and metastasis (5). 

Endocervical adenocarcinoma is the second most commonly cervical cancer in the world (9). Most are HPV-associated (10, 11) and about 15% are HPV-independent (9, 12-15). HPV-independent tumors include gastric, clear cell, mesonephric and endometrioid-type carcinomas, and adenocarcinoma NOS, which have a worse prognosis. Gastric type is more prevalent and is the second most common adenocarcinoma of the cervix (9, 16, 17). These types of tumors mimic other neoplasms in terms of cytology and architecture. Currently, there is no reliable immunohistochemical marker for the diagnosis of gastric-type endocervical adenocarcinoma (9). 

The GATA binding protein 3 (GATA3) is a transcription factor consisting of six members GATA1 to GATA6. These proteins recognize the DNA sequence of nucleotides G-A-T-A in the target gene promoters via two zinc-finger domains (18-20). GATA3 immunohistochemistry has been described as a highly sensitive marker in determining carcinomas of breast and urothelial origin (21, 22). In the gynecologic system, it can be used as a marker to diagnose mesonephric or mesonephric-like carcinomas and trophoblastic tumors (23). Although it is generally negative in tumors of gynecological origin, in some studies, the expression of this marker was shown in 10% of ovarian, 7% of endometrial, and 18% of endocervical tumors. Determination of the prevalence and intensity scores in common female genital tract tumors is important for distinction from mesonephric/-like carcinomas as well as metastatic work-up and excluding carcinomas of breast origin (24). 

Due to the limited number of studies, the present study was performed to determine the diagnostic value of GATA3 in gynecological adenocarcinomas. We especially included a higher number of clear cell carcinomas, as it’s an uncommon subtype of carcinogen in the female genital tract and less focused in the previous studies.

## Material and Methods

This cross-sectional study evaluated 187 samples from different types of endometrial, endocervical, and ovarian carcinoma who underwent biopsy, hysterectomy, and oophorectomy from 2018 to 2021. Due to the rarity of clear cell carcinoma, the specimens with this diagnosis, since three years earlier (2015) were also included. The study was approved by the local ethics committee (number IR. TUMS. IKHC. REC 1401. 075). The slides and paraffin blocks were retrieved from the archive of the pathology department of Imam Khomeini Hospital, Tehran University of Medical Science. A pathologist reviewed the slides, and after confirming the diagnoses, the blocks with adequate tumoral tissue, without or with minimal necrosis or hemorrhage, were selected for immunohistochemical staining. 

Four-μm sections were prepared from the appropriate paraffin blocks. After deparaffinization and rehydration, EDTA buffer was used for antigen retrieval. Following 3-5 times rinse and endogenous peroxidase block (10 minutes at room temperature), incubation with primary antibody (Mouse anti-human monoclonal antibody, MAD-000632QD, Master Diagnostica; Spain) was done. For detection, the Master polymer plus system (Master diagnostic; Spain) was used. After counterstaining with Hematoxylin and mounting, the slides stained with GATA3 antibody were examined by two pathologists independently. Only nuclear staining of tumor cells was considered a positive result. Invasive ductal carcinoma of the breast was considered as a positive control in each run. Also, the H score was determined based on the intensity and percentage of tumor cell staining. The nuclear staining intensity of tumor cells was scored as negative (0), weak (1+), moderate (2+), and severe (3+). The H score was obtained by multiplying the intensity by the percentage of tumor cells stained with GATA3, which ranged from 0 to 300. 

The patient's clinical characteristics, including age and the pathologic information of each sample, such as the histological subtype and pathologic stage, were extracted from the clinical documents and microscopic observation of H&E slides. Recurrence and metastasis status, as well as survival status of patients and overall survival and disease-free survival after surgery, were determined by telephone calls to patients. 

The relationship between GATA3 expression and clinicopathological parameters was investigated. 

After data collection, the analysis of data was conducted with SPSS version 21.0 (SPSS Inc., Chicago, Ill., USA). The results related to qualitative variables were calculated and reported as frequency and percentage, and the results about quantitative variables were calculated as average and standard deviation. Each of the quantitative and qualitative data was statistically analyzed by appropriate statistical tests such as independent t-test, Mann-Whitney, and chi-square test. Also, the Kaplan-Meier curve and Log Rank statistical test were used to investigate the relationship between GATA3 expression and overall survival and disease-free survival. A P-value <0.05 was considered significant.

## Results

This study examined 187 patients including 101 ovarian, 77 endometrial, and 9 endocervical adenocarcinoma samples. The average age was 53.3 ± 12.62 years, with a median age of 54 and a range of 20 to 79 years. The average age of patients with endometrial carcinoma (57.8±11.6) was significantly higher than ovarian (51.1±11.7) and endocervical carcinomas (38.6±6.4). The most common histological subtype observed in ovarian, endometrial, and endocervical tumors was high-grade serous (60.4%), endometrioid (72.7%), and HPV-associated adenocarcinomas, respectively. The frequency of histological subtypes of tumors investigated in this study is shown in Table 1. 

**Table 1:** The frequency of histological subtypes

Histologic Subgroup	Ovarian Carcinoma	Endometrial Carcinoma	Endocervical carcinoma
High-grade serous carcinoma	61 (60.4%)	9 (11.7%)	-
Low-grade serous carcinoma	5 (5%)	-	-
Clear cell carcinoma	19 (18.8%)	12 (15.6%)	-
Endometrioid carcinoma	10 (9.9%)	56 (72.7%)	-
Mucinous carcinoma	6 (5.9%)	_	
Endocervical HPV associated	-	-	7 (77.7%)
HPV independent	-	-	2 (22.3%)

The frequency of each tumor stage in ovarian and endometrial carcinoma samples is shown in Table 2. Samples from endocervical adenocarcinomas were obtained from tumor biopsy in 6 patients. Therefore, the tumor stage and survival cannot be evaluated in these cases. 

**Table 2:** The frequency of tumor stages

Tumor stage	Ovarian Carcinoma	Endometrial Carcinoma
I	27 (27. 3%)	45 (60.8%)
II	5 (5. 1%)	12 (16.2%)
III	65 (65. 7%)	15 (20.3%)
IV	2 (2%)	2 (2.7%)

**Fig 1:**
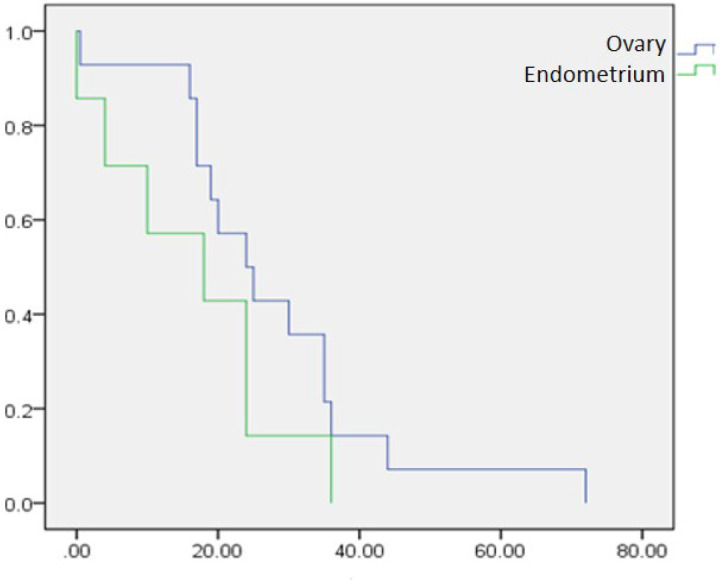
Kaplan-Meier curve. overall survival of patients according to tumor location

Due to the lack of access to some patients, the information related to patient survival in 48 patients with ovarian cancer and 10 patients with endometrial cancer was examined. The survival rate in patients with ovarian carcinoma was significantly higher than in endometrial carcinoma, but there was no significant difference between the two groups regarding metastasis and recurrence. Accordingly, there was no statistically significant difference between overall and disease-free survival between ovarian and endometrial carcinoma (Table 3, Figure 1). 

**Table 3:** Average overall survival and disease-free survival in ovarian and endometrial carcinoma

Tumor type	Average OS (month)	Average DFS (month)
Ovarian Carcinoma	27.89 ±16.8	19. 4± 15.74
Endometrial Carcinoma	16.57 ±12.68	18± 13.52
P-value	0.172	0.475

**Fig. 2:**
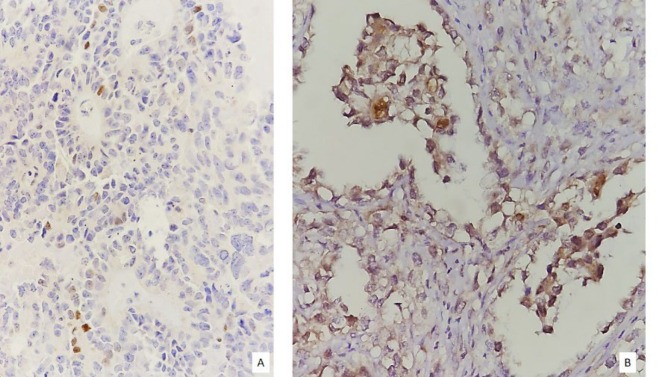
A) Ovarian High-grade serous carcinoma and B) clear cell carcinoma with weak heterogeneous nuclear staining in tumor cells

In an immunohistochemical study, four (5.1%) endometrial endometrioid carcinomas were positive for GATA3, and the expression of this marker was observed in 13 (12.9%) ovarian and 1 (11.1%) endocervical adenocarcinoma. Among the ovarian carcinomas with positive staining, 11 were high-grade serous, 1 endometrioid, and one clear cell carcinoma (Table 4, Figure 2). Also, for cases with positive GATA3 expression, based on the intensity and percentage of tumor cell staining, the H score was determined, which ranged from 2 to 35, with an average of 10.6. 

Due to the negativity of GATA3 expression in most of the endometrial and endocervical adenocarcinoma samples, it was not possible to investigate the relationship between the expression of this marker and the clinical and histopathological features affecting the prognosis as well as the survival status in these patients. These investigations were performed only for ovarian carcinoma samples. The only positive endocervical adenocarcinoma was a case of HPV-associated usual-type adenocarcinoma. There was no significant correlation between patients' age with GATA3 expression in tumor cells (*P*=0.083). There was also no statistically significant correlation between GATA3 expression in tumor cells with clinical stage, chemotherapy response score, and tumor recurrence or metastasis.

**Table 4:** Immunohistochemical expression of GATA3 in tumor cells in different histological subtypes of female genital tract adenocarcinomas (HGSC: high-grade serous carcinoma, LGSC: low-grade serous carcinoma, CCC: clear cell carcinoma, ENDC: endometrioid carcinoma

GATA3 expression in tumor cells				Histologic subtype					
				HGSC	LGSC	CCC	ENDC	MC	Endocervical
Positive	Site	Ovary	Count	11		1	1		
		Endometrium	Count				4		
		Cervix	count	0		0	0		1
Negative	Site	Ovary	Count	50	5	18	9	6	
		Endometrium	Count	7	0	12	40	0	
		Cervix	Count	0	0	0	0	0	8

## Discussion

GATA-binding protein 3 (GATA3) is a transcription factor protein that recognizes G-A-T-A containing sequences in target genes. GATA family members are divided into two subgroups: the first group includes GATA1,2,3, which play a role in the development of the nervous and hematogenous systems (25). The second subgroup includes GATA 4, 5, and 6, which are effective in the development of organs derived from mesoderm and endoderm (26). 

GATA3 is normally expressed in luminal cells of breast ducts and epithelium of the bladder, ureter, and pelvis. Therefore, the immunohistochemical expression of this protein is widely used in the diagnosis of breast and bladder carcinomas and some subtypes of renal cell carcinoma (27). 

In tumors originating from the female reproductive system, the expression of this marker has been proven in trophoblastic tumors and mesonephric carcinomas. However, there have been fewer studies related to its expression in other carcinomas, especially clear cell carcinoma. In the present study, 11 (16.3%) of ovarian high-grade serous carcinomas, 1 (5.5%) of ovarian clear cell carcinomas, and 11.1% of ovarian and 5.1% of endometrial endometrioid carcinomas showed focal and weak staining for GATA3, and the mean calculated H score was 10.6. Only two cases with an H. score of 25 and one case of 35 were identified, which indicates the low prevalence of this marker expression in different subtypes of carcinomas of the female reproductive system. 

In a study by Clark *et al.*, GATA3 expression was investigated by immunohistochemistry in several cytokeratin 7-positive carcinomas, including 55 endometrial carcinomas and 50 ovarian carcinomas. The expression of this marker was determined semi-quantitatively using the H-score method, and a score higher than 10 was considered positive. Based on the results of this study, GATA3 expression was observed in 18% of endocervical, 7% of endometrial, and 10% of ovarian carcinomas, including 7% of serous and 13% of clear cell carcinomas, often with low intensity (H score<50). All 5 ovarian endometrioid adenocarcinomas were negative. The average H score in endometrial and ovarian carcinomas was 37 and 42, respectively. 

 In a study by Terzic et al published in 2019, GATA3 expression was investigated in common gynecological carcinomas. To evaluate GATA3 expression among common gynecological cancers with different histological types, 100 ovarian carcinomas, 64 endometrial carcinomas, 14 endocervical adenocarcinomas, and 16 cervical squamous cell carcinomas were analyzed by immunohistochemical method. Based on the results of this study, 8% of endometrial carcinomas expressed GATA3, which included 2 serous carcinomas, one carcinosarcoma, and one atypical hyperplasia. Of ovarian carcinomas, 6%, including 2 clear cell carcinomas, 2 mucinous adenocarcinomas, and 2 high-grade serous carcinomas, also showed GATA3 expression. The expression of this marker was observed in 38% of cervical squamous carcinomas. All endocervical adenocarcinomas were completely negative. Overall, GATA3 showed a weak to moderate localized expression in a series of endometrial and ovarian carcinomas (13). 

In a study published in 2014, Miettinen and colleagues examined 2500 different epithelial and non-epithelial tumors for GATA3 expression using a monoclonal antibody (clone L50-823) by immunohistochemistry. Based on the results of this study, GATA3 expression was observed in 6 out of 89 cases of endometrial adenocarcinoma (7%) and 4 out of 73 cases of ovarian serous carcinoma (6%). Saber histological types of ovarian carcinoma (25 cases) had no expression of this marker. The percentage of positive tumor cells in these samples was between 5-30%, except for one case of endometrial carcinoma in which 100% of tumor cells were positive (14). 

In Engelsen et al. 's study published in 2008, GATA3 expression was investigated in 316 endometrial carcinomas. Based on the results of this study, GATA3 positive expression in hysterectomy specimens is significantly associated with high clinical stage, serous/papillary and clear cell subtypes, high histological grade, lack of PR expression, aneuploidy, high infiltration, pathological expression of P53 and P16 and poor prognosis. In conclusion, GATA3 expression is associated with an aggressive phenotype of endometrial carcinoma and provides independent prognostic information (9). 

In a study by Liu *et al.* published in 2012, the immunohistochemical expression of GATA3 was investigated in various tumors and normal tissues, including 96 endometrial carcinomas and 56 ovarian serous carcinomas. According to the results of this study, only 2 samples of endometrial adenocarcinoma (2%) showed the expression of GATA3, but the expression of this marker was not observed in any of the ovarian carcinoma samples, as well as normal endometrial and ovarian tissues (10). 

In a study conducted by Lee *et al.* and published in 2023, mesonephric marker expression in low-grade endometrial endometrioid carcinoma was evaluated. It was found that 10.0% of tumors had at least moderate nuclear GATA3 staining. Considering that mesonephric-like adenocarcinoma is in the differential diagnosis of endometrioid carcinoma, the researchers concluded that pathologists should not exclude endometrioid carcinoma based solely on the presence of focal immunoreactivity for mesonephric markers (28). 

Unfortunately, in the current study, there was no mesonephric carcinoma to evaluate the expression of this marker and compare it with other subtypes, which is predictable due to the rarity of this tumor, and studies with a much higher volume are needed to ensure a sufficient number of cases of this subtype.

## Conclusion

GATA3 is infrequently, weak, or focally expressed in most of the common gynecological adenocarcinomas. Ovarian HGSC and endometrial endometrioid carcinoma cells more commonly may be positive for this marker.
